# Eyes-Free Tongue Gesture and Tongue Joystick Control of a Five DOF Upper-Limb Exoskeleton for Severely Disabled Individuals

**DOI:** 10.3389/fnins.2021.739279

**Published:** 2021-12-17

**Authors:** Mostafa Mohammadi, Hendrik Knoche, Mikkel Thøgersen, Stefan Hein Bengtson, Muhammad Ahsan Gull, Bo Bentsen, Michael Gaihede, Kåre Eg Severinsen, Lotte N. S. Andreasen Struijk

**Affiliations:** ^1^Neurorehabilitation Robotics and Engineering, Center for Rehabilitation Robotics, Department of Health Science and Technology, Aalborg University, Aalborg, Denmark; ^2^Human Machine Interaction, Department of Architecture, Design and Media Technology, Aalborg University, Aalborg, Denmark; ^3^Department of Materials and Production, Aalborg University, Aalborg, Denmark; ^4^Department of Clinical Medicine, Aalborg University, Aalborg, Denmark; ^5^Department of Neurology, Aarhus University Hospital, Aarhus, Denmark

**Keywords:** tongue computer interface, upper-limb exoskeleton, rehabilitation robotics, human-robot interaction, disabled individuals, assistive devices, tetraplegia

## Abstract

Spinal cord injury can leave the affected individual severely disabled with a low level of independence and quality of life. Assistive upper-limb exoskeletons are one of the solutions that can enable an individual with tetraplegia (paralysis in both arms and legs) to perform simple activities of daily living by mobilizing the arm. Providing an efficient user interface that can provide full continuous control of such a device—safely and intuitively—with multiple degrees of freedom (DOFs) still remains a challenge. In this study, a control interface for an assistive upper-limb exoskeleton with five DOFs based on an intraoral tongue-computer interface (ITCI) for individuals with tetraplegia was proposed. Furthermore, we evaluated eyes-free use of the ITCI for the first time and compared two tongue-operated control methods, one based on tongue gestures and the other based on dynamic virtual buttons and a joystick-like control. Ten able-bodied participants tongue controlled the exoskeleton for a drinking task with and without visual feedback on a screen in three experimental sessions. As a baseline, the participants performed the drinking task with a standard gamepad. The results showed that it was possible to control the exoskeleton with the tongue even without visual feedback and to perform the drinking task at 65.1% of the speed of the gamepad. In a clinical case study, an individual with tetraplegia further succeeded to fully control the exoskeleton and perform the drinking task only 5.6% slower than the able-bodied group. This study demonstrated the first single-modal control interface that can enable individuals with complete tetraplegia to fully and continuously control a five-DOF upper limb exoskeleton and perform a drinking task after only 2 h of training. The interface was used both with and without visual feedback.

## 1. Introduction

The estimated incidence of spinal cord injury (SCI) is between 250 000 and 500 000 worldwide (Bickenbach et al., 2013). The highest incidence rate is found within young adults (20–29 years for males; 15–19 years for females) (Bickenbach et al., 2013) with a median survival time of 38 years after the injury (McColl et al., 1997). SCI in the cervical levels of the spine affects both the upper and lower body (so-called tetraplegia) and leads to partial or complete loss of voluntary control of both arms and legs, which accounts for approximately one-third of SCI cases (Wyndaele and Wyndaele, 2006). Individuals with tetraplegia usually require full-time assistance with the activities of daily living (ADLs) and desire to increase the level of dependence (Manns and Chad, 2001). Therefore, restoration of the arm functionality remains critical and highly prioritized to improve the quality of life and independence.

Assistive upper-limb exoskeletons (ULEs) are robotic devices that augment human muscles or substitute for the lost functionality or weakness in the arm in individuals having suffered a stroke or SCI. These technologies can improve the users' quality of life by facilitating more independence and autonomy in the ADLs. The number of actuated joints of a ULE varies from a single degree-of-freedom (DOF) (Tang et al., 2014; Crea et al., 2017; Hosseini et al., 2017) to seven DOFs including shoulder, elbow and wrist joints (Barsotti et al., 2015; Cui et al., 2016; Kim and Deshpande, 2017). Major challenges in many of the proposed assistive ULEs are their bulkiness, and being limited to a fixed setup; i.e., lack of mobility. The EXOTIC exoskeleton (EXOTIC Exo) is a five-DOF ULE designed to assist individuals with tetraplegia to perform prioritized ADLs (Thøgersen et al., 2020) such as drinking or eating snacks (Kobbelgaard et al., 2021). Further, it can be attached to the user's wheelchair.

ULEs are challenged by the lack of efficient high-level control techniques for individuals with complete tetraplegia who may be the most in need. The majority of the developed ULEs require some residual levels of arm movement or contraction in the arm muscles for the control (Miao et al., 2018; Gull et al., 2020). For example, the impedance/admittance control schemes operate based on minimal motions of the user, which is followed and augmented by the exoskeleton (Bai et al., 2017; Kim and Deshpande, 2017). Gandolla et al. (2021) used a finger-controlled sensitive joystick and a voice control interface to control a four-DOFs ULE. Another common approach relies on the remaining volunteer control of the arm muscles and uses electromyography (EMG)/force myography (FMG) sensors for detecting the muscle contraction in order to identify the intention of the user to move the arm (Tang et al., 2014; Hosseini et al., 2017; Islam and Bai, 2017). However, these systems are not applicable in severe or complete tetraplegia.

Brain-computer interfaces (BCI) have the potential to enable individuals with complete tetraplegia to control a ULE. Sakurada et al. (2013) adapted a method for identifying visually evoked potentials (VEP) from electroencephalography (EEG) signals to initiate motion of a ULE from a set of six pre-defined movements, which were recorded in the system memory beforehand. Further, it is possible to detect the intention of the user to move the arm from motor imaginary (MI) potentials (Barsotti et al., 2015; Brauchle et al., 2015) or movement-related cortical potentials (MRCPs) (Bhagat et al., 2016) extracted from EEG. However, MI and MRCP interfaces only triggered pre-defined motions. Incorporating BCI with other input modalities such EMG, electrooculography (EOG), and eye-tracking improves the lack of sufficient control commands (Soekadar et al., 2016). Nann et al. (2021) implemented a control interface for controlling a six-DOF ULE based on EEG and EOG signals. Frisoli et al. (2012) used a control scheme based on computer vision, eye-tracker, and an MI BCI. The computer vision and eye-tracker detected the intended object, and the MI BCI initiated actions toward the object (Frisoli et al., 2012). BCIs are highly beneficial for individuals with a locked-in syndrome that cannot use other interfaces. However, none of the aforementioned BCI setups afforded completion of an arbitrary task or full manual continuous control of the multi-DOF ULE due to the lack of sufficient input commands. A **continuous control** through which the user possesses the control at all instances and contrary to discrete control that the user only initiates a movement and the system continues without user involvement allows for fine control and ensures higher safety. Another characteristic of some of these interfaces, e.g., systems based on SSVEP and eye-tracking, is the need for constantly looking at a screen. This indirect way of control through a screen may distract the focus during the control and thus degrade the performance (Bragdon et al., 2011) and increase the collision risk. Using computer vision for automatic grasping can reduce the grasping time. However, users may prefer no automation in order to possess more flexibility and freedom in the control (Kim et al., 2012).

Some studies that incorporated invasive BCIs demonstrated the possibility of continuous high-dimensional control for individuals with tetraplegia (Wang et al., 2013; Wodlinger et al., 2014; Benabid et al., 2019). Benabid et al. (2019) implanted two epidural recorders, each with 64 electrodes, above the sensorimotor cortex of an individual with tetraplegia and enabled him to control eight DOFs of an exoskeleton after 16 months from implanting. Wodlinger et al. (2014) showed the possibility of controlling a 10-DOF prosthetic limb using two 96-channel intracortical electrode arrays implanted in the left motor cortex. Invasive BCI approaches are still very limited due to the high cost, time-consuming (several months) and unstable calibration, high risk, and uncertain clinical compatibility.

The intraoral tongue-computer interface (ITCI) (Struijk et al., 2017) provides 18 input commands and has a commercially available version for individuals with tetraplegia (ITongue, TKS). An assistive robotic manipulator with seven degrees of freedom was previously interfaced with the ITCI and enabled a participant with complete tetraplegia to perform tasks with the robot (Andreasen Struijk et al., 2017b). Another tongue interface for a ULE was presented by Zhang et al. (2021) that provided control over two DOFs of a rehabilitation ULE for a tracking task.

In previous studies using the ITCI, virtual buttons with a visual feedback on a screen were utilized to interface an electronic device (Andreasen Struijk et al., 2017b; Mohammadi et al., 2021). However, the role of the visual feedback and the effect of removing it from the setup were not investigated. The need for a screen remains a persisting challenge in current interfaces for individuals with tetraplegia, including brain and eye-based interfaces. The attention demand differs between virtual buttons and gestures in touch input devices (Bragdon et al., 2011), and for a mobile phone, gestures are less attention demanding (Bragdon et al., 2011). Bragdon et al. (2011) compared virtual buttons with several gestural input techniques in the presence of some environmental distraction. Not looking at the screen reduced people's performance with virtual buttons but not with gestures (Bragdon et al., 2011). Furthermore, some studies found that people can perform gesture-based touch input interactions eyes-free (Pirhonen et al., 2002; Kubo et al., 2016).

To address the current challenges with insufficient contentious commands in robot interfaces for individuals with severe tetraplegia, and with the current need for visual feedback as part of the interface compromising the robot control, this study for the first time explores eyes-free tongue control of a five-DOF ULE; the EXOTIC Exo. We compared two control methods: one based on tongue gestures and the other based on dynamic virtual buttons and a joystick-like control. The two control methods were used both with and without visual feedback.

## 2. Methods

### 2.1. Participants

Ten able-bodied volunteers (mean age 24.7, range 19–34, one female) participated in this study. None of them had prior experience with the ITCI or a ULE, and none of them were students or employees in the authors' departments. All the participants received written and oral information about the study and the risks, and they signed consent forms before participating.

Following the experiment with the able-bodied cohort, a potential user of the EXOTIC Exo voluntarily participated in a case study for evaluation of the system. He was 23 years old and had suffered an incomplete SCI at the C2 level 9 months prior to the experiment. A medical doctor assessed the impairment level using the International Standards for Neurological Classification of SCI (ISNCSCI) and issued a “C” on the ASIA impairment scale and a total upper-extremity motor score (UEMS) of 6, i.e., a total paralysis of the whole arm except residual viable contraction in wrist extensors and elbow flexors. The user was dependent on a ventilator for breathing and could not use his arms and fingers for controlling his wheelchair and other ADLs.

The local ethical committee had approved the study with approval number N-20190030 for able-bodied participants and N-20210016 for the user study.

### 2.2. System Overview

#### 2.2.1. EXOTIC Upper-Limb Exoskeleton

**Figure 1:**
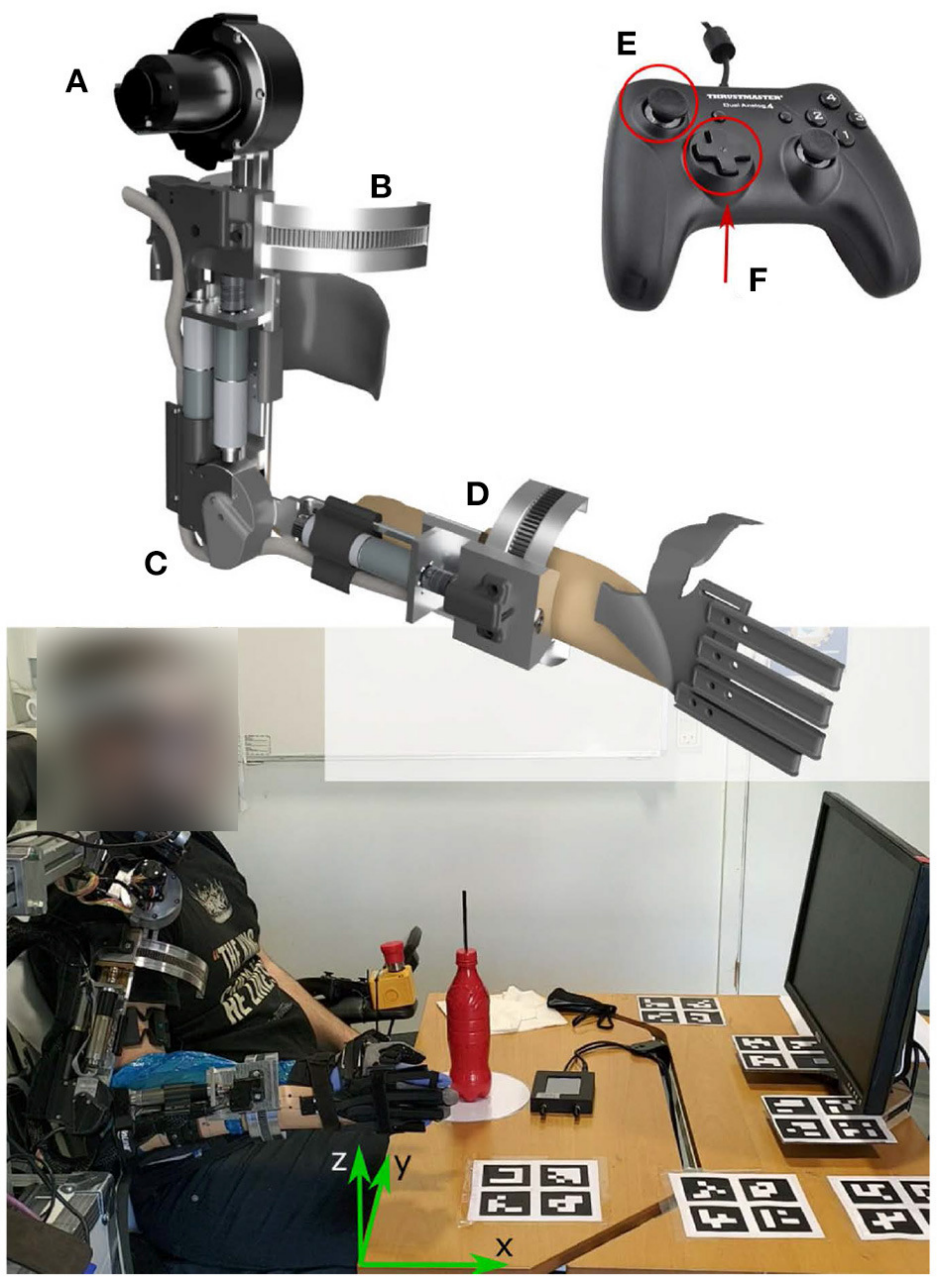
Top-left: The EXOTIC Exo DOFs consisted of **(A)** shoulder flexion/extension, **(B)** shoulder internal/external rotation, **(C)** elbow flexion/extension, **(D)** wrist supination/pronation, and hand opening/closing. Top-right: The gamepad 2D joystick **(E)** controlled the hand position in the X-Y plane, and the four directional buttons **(F)** moved the hand up and down and rotated the wrist. Bottom: Participants seated on a wheelchair in front of a screen. An eye-tracker goggle tracked the gaze direction. The ChArUso markers on the table were used to find the transformation between the gaze direction and the exoskeleton coordinate frames.

The EXOTIC Exo (Figure 1, top-left) was designed based on user preferences and desires obtained through interviews and design games with target users, i.e., individuals with tetraplegia (Kobbelgaard et al., 2021). The five actuated DOFs included shoulder flexion/extension, shoulder external/internal rotation, elbow flexion/extension, wrist supination/pronation, and hand open/close. In the first four joints, the torque was transmitted from a motor to the joint through gear mechanisms, and an absolute encoder measured the joint angle for closed-loop control (Figure 1, top-left). The EXOTIC Exo arm was combined with the CarbonHand (BioServo, Sweden) to move the user's fingers and close the hand for grasping objects. A custom-designed hand brace opened the hand using elastic bands. We implemented the control algorithm in the Robot Operating System (ROS kinetic), which handled the processing and communication between the different modules of the system. Further details of the EXOTIC Exo design are available in Thøgersen et al. (2020).

The participants controlled their right hand velocity in a fixed Cartesian coordinate frame (see green arrows in Figure 1, bottom), such that the x-axis was perpendicular to the user frontal plane, the y-axis was perpendicular to the sagittal plane, and the z-axis was perpendicular to the horizontal plane. We set the hand velocity to 4.5 cm/s based on pilot tests to ensure a smooth and safe control. The hand (end-effector) velocity commands from the ITCI were transformed to joint velocity commands using the kinematic model of the exoskeleton (Gull et al., 2021) and an inverse-kinematics algorithm (Ruppel, 2017).

#### 2.2.2. Intraoral Tongue-Computer Interface

**Figure 2:**
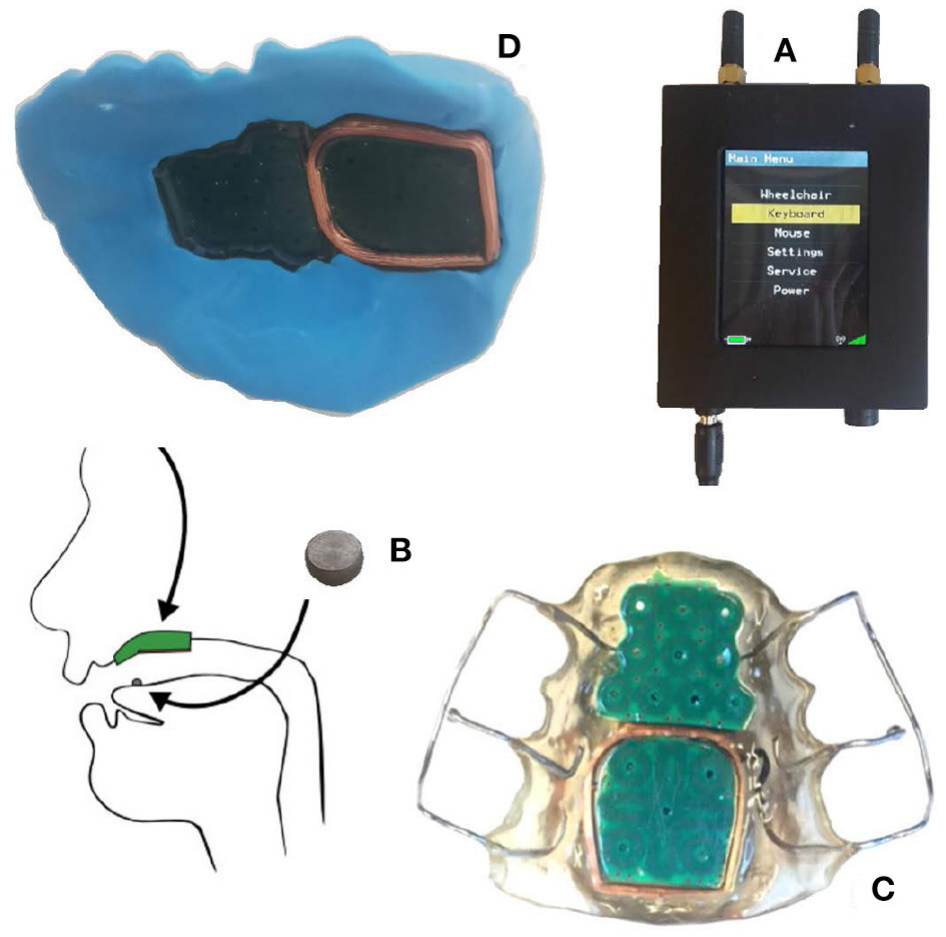
The ITCI system consisted of **(A)** the central unit, **(B)** the activation unit, and **(C)** the mouthpiece unit. **(D)** Shows the temporary mouthpiece unit made from silicon putty.

The participants controlled the EXOTIC Exo using an ITCI. The ITCI (Figure 2) consists of a mouthpiece unit (MPU), an activation unit (AU), and a central unit (CU) (Struijk et al., 2017). The MPU is a dental retainer encompassing 18 inductive sensors arranged in two printed circuit boards (PCB). The AU is a cylindrical titanium alloy (5 mm in diameter and 3 mm height) that is either glued or pierced to the tip of the tongue. The vicinity of the AU to the inductive sensors applies a voltage variation over the sensors that is amplified, rectified, and low-pass filtered by the embedded electronics in the MPU (Andreasen Struijk, 2006) and then transmitted over wireless communication to the CU at 30 Hz. The CU receives the sensors data and emulates a joystick for a wheelchair or a mouse and keyboard for a computer (Andreasen Struijk et al., 2017a).

Unlike the commercial version, we created a temporary dental retainer (Figure 2D) for each participant using dental putty (ImpressA Putty, TopDent). ImpressA consists of two soft putties that solidify about 2 min after mixing them. Immediately after mixing the two putties, we embedded the ITCI MPU into the putty and gently pressed it toward the participant's palate. After 2 min, we took out the resulting mouthpiece and trimmed the residuals with a scalpel. The fine impression of the uneven palate surface and the cavities around the teeth made a suction effect that prevented the mouthpiece from falling out after it was placed at the palate. The able-bodied participants could easily take out the mouthpiece and later remount it inside their mouth as needed. The putty-based retainer had a bigger size and a weaker signal (due to a shorter antenna) than the standard commercial acrylic retainer (Figures 2C,D). However, the putty retainer had an acceptable performance and was easier to produce as no plaster mold of the upper mouth had to be produced prior to the experiment. We glued the AU on the participant's tongue approximately 1 cm posterior to the tip of the tongue using Histoacryl (B.Braun Surgical S.A., Spain) to enable activation of the mouth piece.

### 2.3. Control Layouts

**Figure 3:**
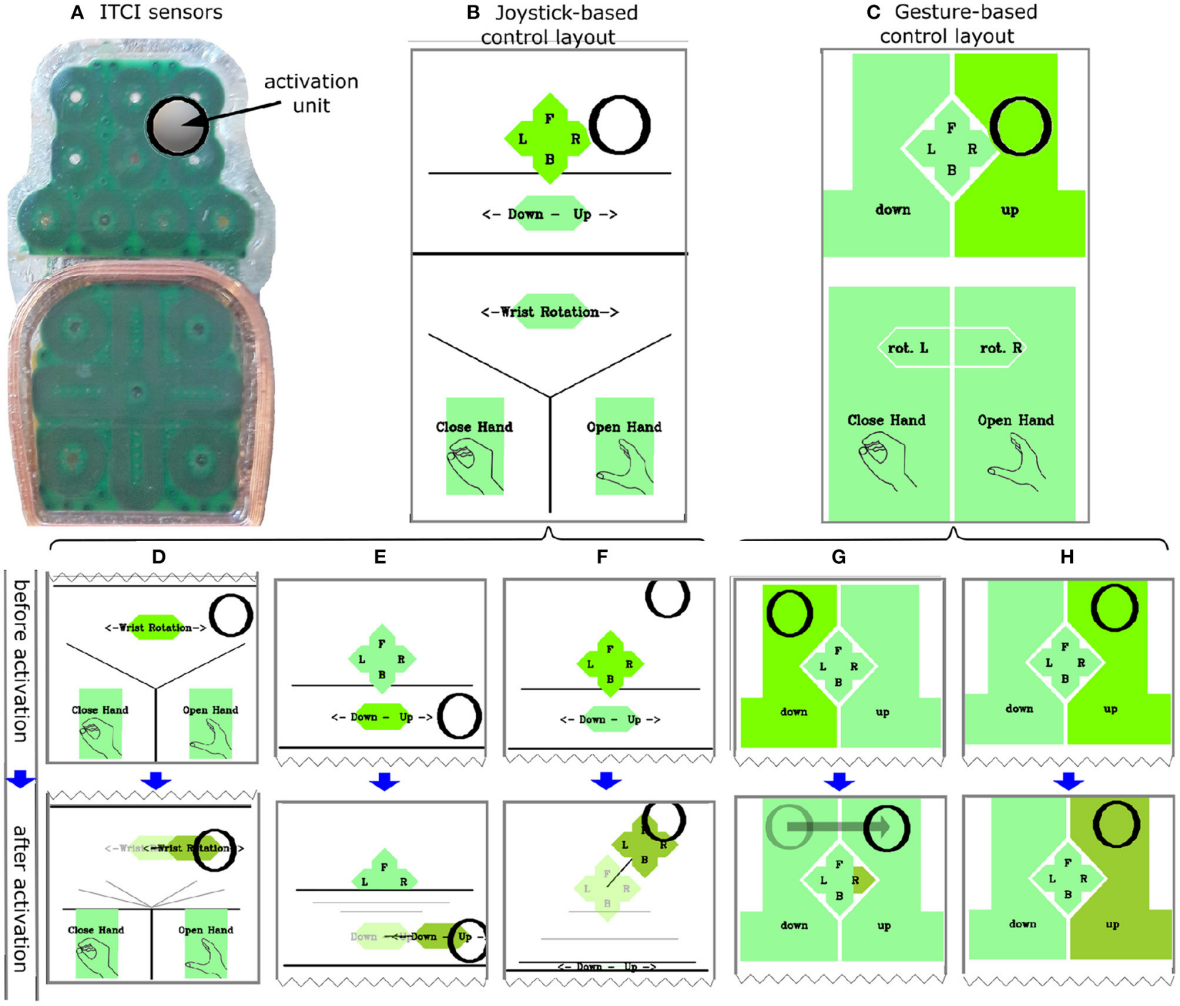
**(A)** The physical ITCI sensors layout and an activation unit in contact with the sensors. The Joystick-based **(B)** and Gesture-based **(C)** control layouts including the black circle as visual feedback for the position of the activation unit. **(D–H)** Behavior of controls before and after activation in which the light green (before activation, top) turns into darker green after the dwell time of 0.5 s (bottom). **(D–F)** Change of dynamic button size after selecting. **(G)** Swipe-press to the right. **(H)** Selecting UP command through dwell.

We developed a control interface software in ROS that sampled the user input at 30 Hz (the data rate from the ITCI) and sent control commands to the exoskeleton's motors at 100 Hz. The software read the raw signals of the 18 sensors (Figure 3A) that were sent from the CU on a serial port and estimated the AU position on the sensor PCBs with approximately 1 mm accuracy using the Weighted Average of Neighbor Sensors method (Mohammadi et al., 2019). Modeling the ITCI PCBs as two small touchpads, we developed a joystick-based and a gesture-based control layout to map the AU position to control commands. These two control layouts provided ten input commands to enable full manual and continuous control of the exoskeleton's five DOFs. The software visualized the control layout and the current position of the AU in contact with the ITCI PCBs on a screen in front of the participants (Figure 1).

#### 2.3.1. Joystick-Based Control Layout

The joystick-based control layout consisted of five virtual controls that were activated by placing the AU on the control area (Figure 3B). Previous studies with ITCIs used virtual buttons with a fixed size and control layouts with two modes to overcome the challenge of pointing to small buttons (Mohammadi et al., 2021). However, mode switches take time and may cause confusion (Herlant et al., 2016). In this study we used dynamic virtual controls that increased in size during selection in order to accommodate all control commands in one control mode, as well as to provide enough large buttons for an easy manipulation (Figures 3D–F). The layout consisted of:

1. A 2D joystick-like control for continuous control over direction and velocity of the hand in a horizontal plane (Figure 3D).
2. A lever-like control for continuous velocity control of the hand in the vertical direction (z-axis) (Figure 3E).
3. A lever-like control for the wrist rotation (supination/pronation) (Figure 3F).
4. Two virtual buttons for opening and closing the hand.

We used a joystick-like control with the ITCI as it improved the performance of a robot interface compared with button-like control commands (Mohammadi et al., 2021). Furthermore, the exoskeleton started moving 0.5 s after the AU had contacted the sensors to avoid unintended commands based on a previous study (Struijk et al., 2018) (Figures 3D–H, top figures). Removing the AU from the MPU immediately stopped all movements of the exoskeleton. The field color switched between three different shades of green to signify whether a control was idle, waiting to complete the dwelling time (no exoskeleton movement), or had completed the dwelling time and was controlling movements of the exoskeleton (Figures 3E–I).

#### 2.3.2. Gesture-Based Control Layout

We developed the gesture-based control layout using both virtual buttons and tongue gestures. Recognizing tongue gestures from the ITCI provides control inputs in addition to virtual buttons. The difference between virtual buttons and gestures lies in the static mapping between contact points and their control commands for virtual buttons and the recognition of contact point movement patterns and kinematic features for tongue gestures. A pilot study previously introduced a tongue gesture recognition method that classified a set of six tongue gestures with 94.3% accuracy (Mohammadi et al., 2020). We used a set of five gestures: swipe-press left, swipe-press right, swipe-press forward, swipe-press backward, and press for developing the gesture-based layout. A press was recognized when the AU touched the ITCI PCB for longer than a dwelling time of 0.5 s without moving further than 4 mm from the initial contact point. A swipe-press was recognized when the AU contact point displaced more than 4 mm in an interval of less than 0.5 s (Mohammadi et al., 2020). The control layout consisted of:

1. Swipe-press gestures on the anterior touchpad of the ITCI moved the exoskeleton hand toward the gesture direction including forward, backward, left, and right (Figure 3G).
2. Swipe-press to the right and left on the posterior touchpad rotated the wrist clockwise and counterclockwise (Figure 3C: rot. L/R).
3. Press gesture within the depicted areas in Figure 3C was used for moving the hand up and down, and for opening and closing the hand (Figure 3H).

Similar to the joystick-based layout, the color-coding illustrated the state of each command (idle, waiting for dwell time completion, activated).

### 2.4. Tasks

#### 2.4.1. Drinking Task

We selected a drinking task due to its high desirability by individuals with tetraplegia (Kobbelgaard et al., 2021). The task started when the exoskeleton was in the home position, similar to mounting the right hand on the wheelchair armrest (Figure 1). The participants controlled the EXOTIC Exo with the ITCI to grasp a bottle filled with 200 mL water. The bottle had a straw of 10 cm long above the bottle lid. To simulate drinking from the bottle, they made contact between the straw tip and a transparent face shield that they wore during the experiment (to avoid accidentally poking the participant eyes with the straw). Finally, they put the bottle back on the table within a circular area with a 20 cm diameter (Figure 1). If the participants dropped the bottle, we would restart the trial.

#### 2.4.2. Button Task

**Figure 4:**
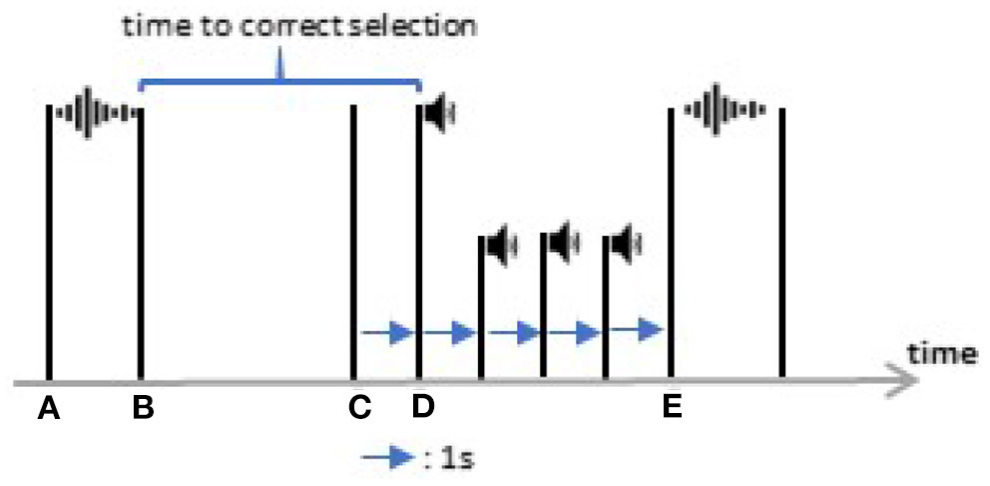
A piece of the button task timeline between two target commands. At **(A)** the computer started to call a new command and at **(B)** the audio (for example “forward”) finished. At **(C)** the correct command was issued and after 1 s **(D)**, the computer approved the target command by a beep sound. After three beeps, a new target command was presented **(E)**.

The second task aimed to investigate the difference between the control commands used in the layouts regarding the time to select and the number of fault commands. The task consisted of issuing all ten commands (up, down, left, right, forward, backward, rotate left, rotate right, open hand, close hand), which were presented to the participant with an auditory cue. With five repetitions of each command, 50 commands were presented in random order. After presenting the target command, the software waited until the participant selected and sustained the target command for 1 s and then notified the participant with a beep sound. We set a 4-s pause before presenting a new target command to relax the tongue. During this pause, three beeps with an interval of 1 s between them notified the participants that a new command would be presented (Figure 4).

### 2.5. Outcome Measures

To investigate the role of the visual feedback from the AU position on the control layout and the effect of removing it, we asked the participants to perform the tasks in two conditions; one with the screen that showed the visual feedback in front of the user and one without it (by turning off the screen). To investigate the visual attention of the participants while using the two layouts with and without visual feedback, the participants wore a monocular eye-tracker headset (Pupil Labs, Germany) consisting of a scene camera that captured the participant's field of view and an eye camera that captured the pupil. We used ChArUso markers (Figure 1) to find the transformation matrices from the table coordinate frame to the eye-tracker frame to identify an intersection between the gaze line and the screen and calculated the fraction of the trial during which their gaze was on the screen (*Gaze-to-screen*). We calibrated the eye-tracker using the Pupil Core (version 3.2) software for Linux at the beginning of the experiment and whenever the headset was displaced.

During all trials, we recorded the user inputs from the ITCI and the gamepad, the exoskeleton joint angles, the hand position (obtained from the joint angles using robot kinematics).

For the drinking task, we obtained the *Task Time* by measuring the interval between the first issued command by the participant and the moment when the glove was opened for releasing the bottle in the target area. *Moving Time* represented the duration in which the exoskeleton was moving, and we defined the remainder as *Pause Time*. Further, we recorded the *Trajectory Length* that the wrist moved and the *Number of Commands* the participants issued.

For the button task, a *Time-to-select* was measured as the interval between the end of the auditory cue and when the target command was issued (Figure 4). The *Number of Fault Commands* during this interval was also recorded.

We used two questionnaires to obtain the participants opinion: one to measure the task load (NASA TLX, Hart, 2006) and the other to measure the intuitiveness of the control (INTUI, Ullrich and Diefenbach, 2015). Higher TLX scores indicate higher task loads, and higher INTUI scores indicate higher intuitiveness. Furthermore, the participants stated whether they preferred the task with or without the visual feedback and which layout they preferred with and without visual feedback being present after the four conditions.

In total, we obtained the following outcome measures for the drinking task:

1. Task time (moving time + pause time)
2. Number of commands
3. Trajectory length
4. Gaze-to-screen (only in the condition with the screen)
5. NASA TLX
6. INTUI

and for the button task:

1. Time-to-select
2. Number of fault commands

### 2.6. Study Protocol and Procedure

#### 2.6.1. Main Study

**Figure 5:**
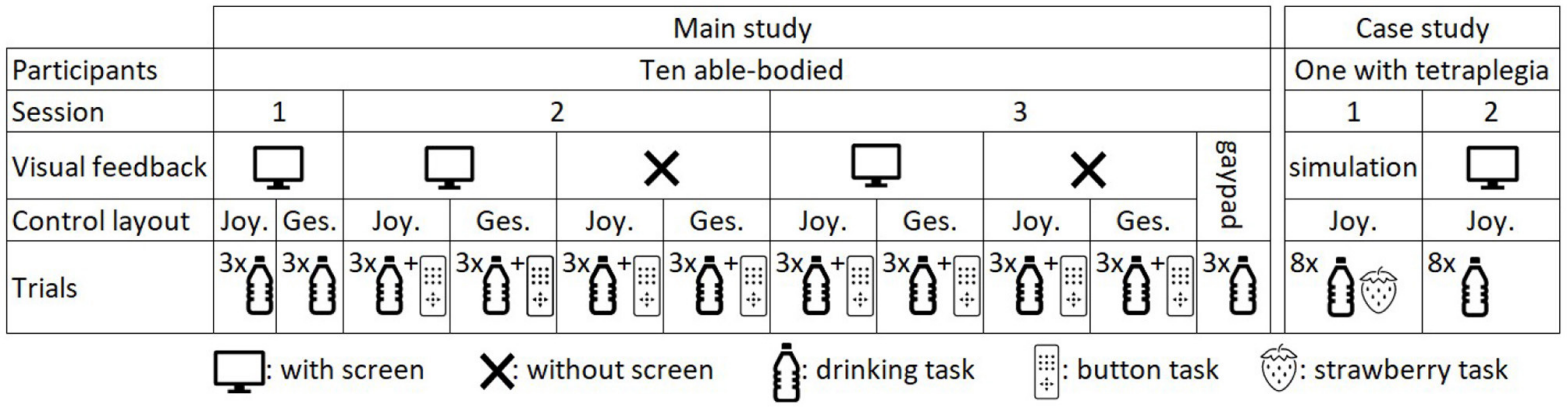
Summary of the experimental sessions, task conditions (with/without screen, Joystick-based/Gesture-based control layout), and the number of repetition.

The experiment for the able-bodied participants consisted of three sessions on 3 consecutive days, each lasting no more than 4 h (Figure 5). During the first session, we made a custom mouthpiece for the participant using the core electronics of the ITCI and the dental putty (Figure 2D). We adjusted the length of the EXOTIC Exo upper arm and forearm based on the participant's anthropometric measures. The participants donned a Carbonhand glove that matched their hand size (S, M, or L) and inserted their arm into the EXOTIC Exo braces. We adjusted the position and height of the exoskeleton such that the participant felt comfortable. A Velcro strap fixed the participant's wrist to the wrist brace, and the upper arm and the forearm braces carried the arm without any straps. Then we placed a table in front of the participant with a screen providing the visual feedback of the control layout (Figure 1). The positions of the wheelchair and the table were fixed and similar for all participants.

In the first session, we used the first 2 h to introduce the study to the participant, create a custom mouthpiece, and adjust the exoskeleton length. We only employed the drinking task to let the participant learn the layouts and the EXOTIC Exo motions while the visual feedback was presented on the screen. The participants trained each control layout by successfully finishing the task once and then repeated the task three times. In the second session, the participants used the two control layouts, both with and without the screen, leading to four conditions in total (Figure 3). In each condition, we recorded one trial of the drinking task as training followed by three repetitions of the drinking task and finally one trial of the button task. The third session was similar to the second session. In addition, the participants filled in NASA TLX and INTUI questionnaires after each condition The order of testing the four conditions was counterbalanced over the participants. The participants had breaks between the trials whenever they desired.

To provide a baseline for the control interface and enable between-study comparisons, at the end of the third session we asked the participants to perform the drinking task with a standard gamepad (Dual Analog 4, Thrustmaster) mounted on the left wheelchair armrest (Figure 1). Like the joystick-based layout, a 2D joystick provided control of the EXOTIC Exo in a horizontal plane. The four directional buttons provided up, down, rotate-left, and rotate-right commands (Figures 1E,F), and button-2 and button-3 afforded opening and closing the hand. We recorded four repetitions of the drinking task (the first one as training) followed by NASA TLX and INTUI questionnaires.

#### 2.6.2. Case Study With a User

**Figure 6:**
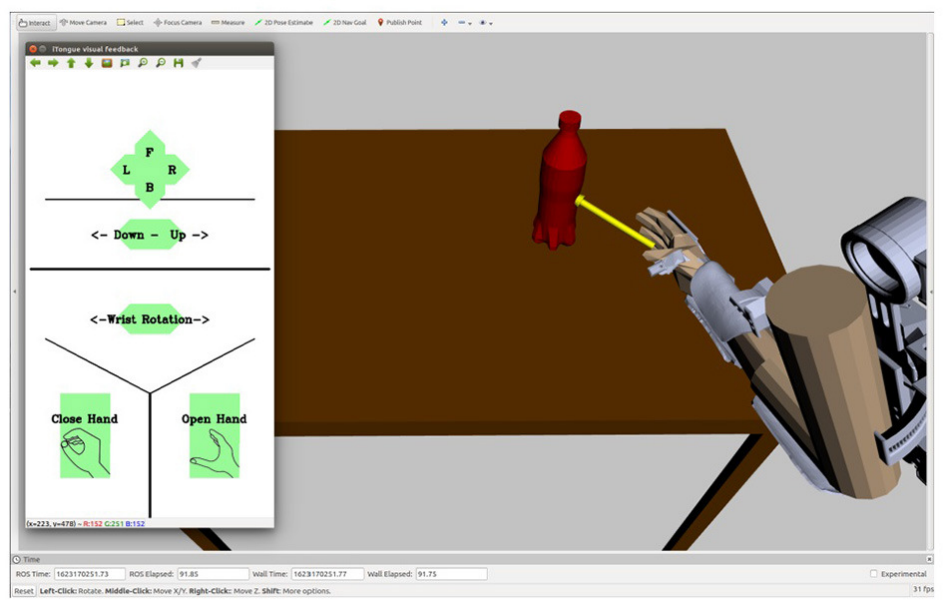
A simulation of the EXOTIC Exo and the drinking task that was used by the user for training in the first session without donning the exoskeleton. The ITCI visual feedback was presented on the left side of the screen.

The case study aimed at demonstrating clinical use of the tongue-exoskeleton interface and not comparing the control layouts in different setups. Therefore, the participant only used the joystick-based layout with the visual feedback because the able-bodied participants achieved the highest performance with that combination. The study consisted of two sessions on consecutive days and was performed at the Spinal Cord Injury Centre of Western Denmark. The user received information about the experiment in a meeting before the experiment and in the first session before starting. Afterwards, we made a custom-made mouthpiece for him. In the first session, the user practiced the ITCI for 2 h by controlling a simulation of the EXOTIC Exo that we presented on a screen in front of the user (Figure 6). He performed eight repetitions of four different tasks including grasping a bottle, grasping a strawberry, grasping a bottle and moving it toward his face, and grasping a strawberry and moving it toward his face. In the second session, we attached the EXOTIC Exo to the user's right hand and asked him to train tongue control of the exoskeleton by completing the drinking task. Afterwards, we recorded eight repetitions of the drinking task similar to the able-bodied group (Figure 5). We recorded the task time, number of commands, and the trajectory length during the case study.

### 2.7. Statistical Analysis

We compared the two control methods (joystick-based and gesture-based) for the drinking task in the two conditions (with and without the screen) using two-way ANOVA and a significance level of 0.05 with a Sidak correction for multiple comparisons. We used Shapiro-Wilk's test to check if the outcome measures were normally distributed. In the case of non-normal distribution, we used a log-transformation and then tested the normality again.

## 3. Results

### 3.1. Tongue Control of the Exoskeleton for Drinking

**Figure 7:**
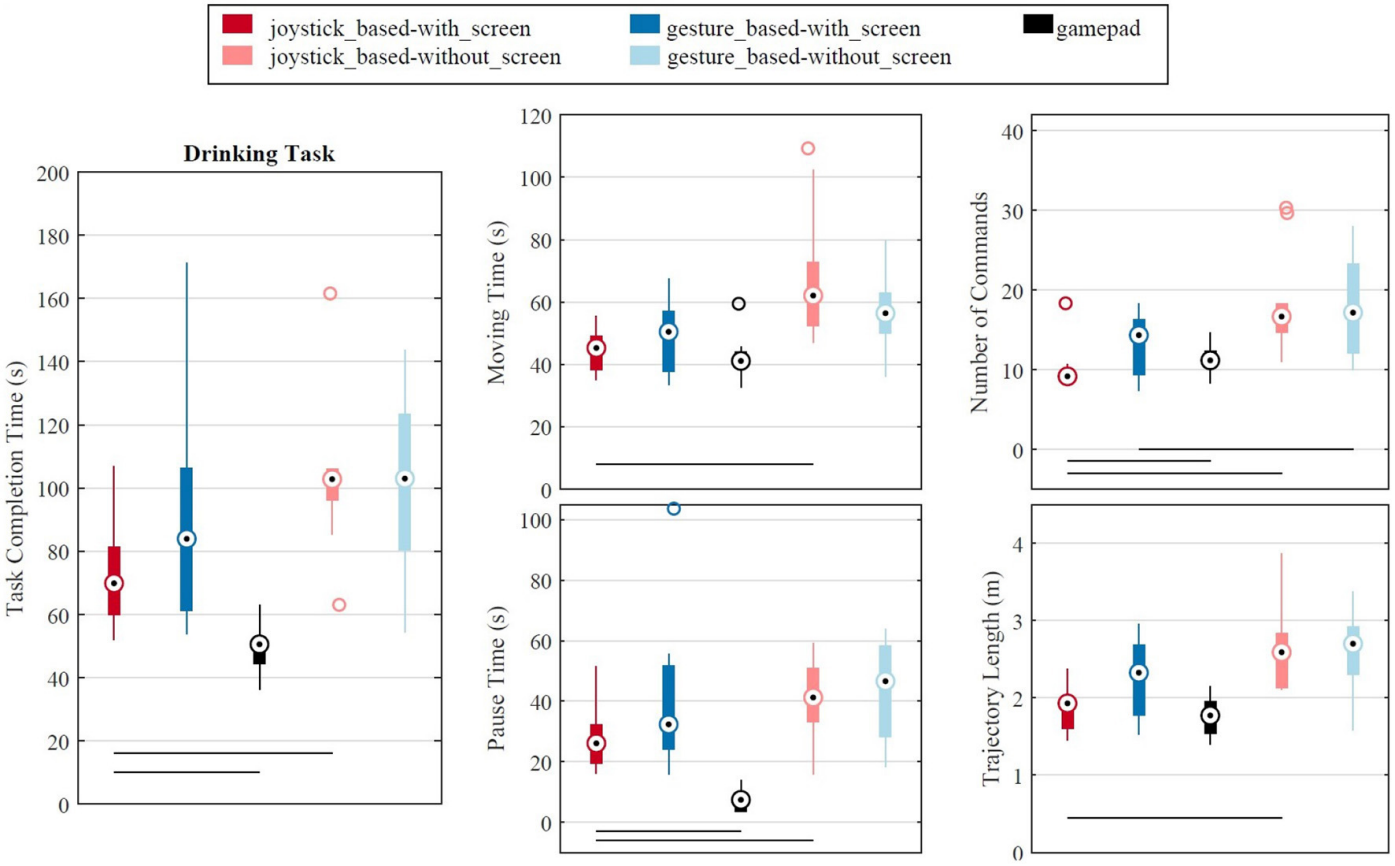
Performance of different control methods for drinking task in the third session. A horizontal line bellow the box blots shows a significant difference between the two conditions that the line connects. The “+” points are the outliers. Bottom-right: The hand trajectory from the initial position to the bottle position is shown with the light green to the dark green points. The position points change color from blue to red on the path from the grasp position to the final hand position.

All participants successfully tongue controlled the EXOTIC Exo for the drinking task (see the Supplementary Material for a video from the drinking task). There was no statistically significant interaction between the effects of control layout (joystick-based vs. gesture-based) and visual feedback (with vs. without) on task time, moving time, pause time, number of commands, and trajectory length (Table 1). The two control methods performed with no significant difference (two-way ANOVA, Table 1) in all the outcome measures (Figure 7).

**Table 1:** The two-way ANOVA and the simple main effect analysis results.

	**Test of within-subjects effects**			**Main effect of screen**	
	**Layout**	**Screen**	Screen* layout	**Joystick-based**	**Gesture-based**
	*F*_(1, 36)_ [*p*]	*F*_(1, 36)_ [*p*]	*F*_(1, 36)_ [*p*]	*F*_(1, 18)_ [*p*]	*F*_(1, 18)_ [*p*]
Task time	0.028 [0.868]	7.940 [**0.008**]	2.128 [0.153]	9.145 [**0.005**]	0.924 [0.343]
Moving time	0.446 [0.508]	13.211 [**0.001**]	2.355 [0.134]	13.361 [**0.001**]	2.205 [0.146]
Pause time	0.713 [0.404]	4.135 [**0.049**]	1.352 [0.253]	5.108 [**0.030**]	0.379 [0.542]
Num. of commands	1.333 [0.256]	18.972 [**0.001**]	2.110 [0.155]	16.867 [**0.001**]	4.215 [**0.047**]
Trajectory length	1.049 [0.312]	11.798 [**0.002**]	1.642 [0.208]	11.121 [**0.002**]	2.319 [0.137]
NASA TLX	0.087 [0.770]	3.670 [0.063]	0.230 [0.634]	-	-
INTUI	0.922 [0.343]	0.583 [0.450]	0.023 [0.881]	-	-

*The test of within-subjects effects showed that the screen had a significant effect on all the performance measures. Thus, we tested the main effect of screen on the two control schemes (right column). Bold p-values show a significant effect of the factor*.

Removing the ITCI visual feedback from the setup produced a statistically significant effect on all performance measures (two-way ANOVA, Table 1), leading to significantly longer task times (45.3%), moving times (35.9%), pause times (53.7%), longer trajectory lengths (31.7%, and more commands (72.2%) while the exoskeleton was controlled using the joystick-based layout (Figure 7). In contrast, removing the visual feedback while using the gesture-based layout did not affect the performance measures, except for the number of commands that increased for 20.1% (Figure 7 and Table 1).

**Figure 8:**
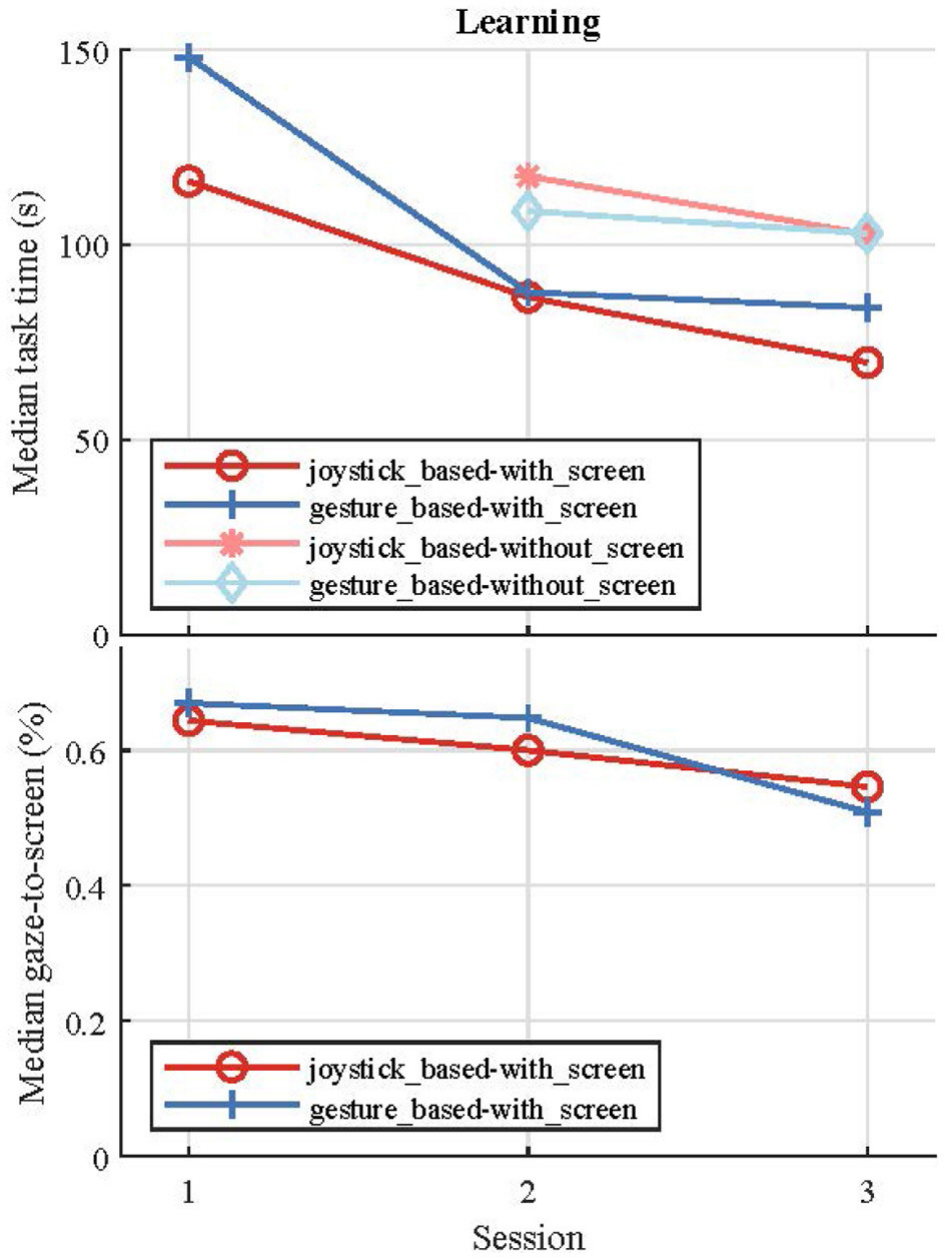
Variation of the drinking task time (top) and the looking to screen fraction over the three experimental sessions.

The task time significantly decreased (one-way ANOVA) from the first session to the second session (Figure 8) for both joystick-based (25.6%, *p* = 0.007) and gesture-based (40.7%, *p* = 0.002) layouts. However, no significant difference in task time was observed from the second to the third session. We observed no significant difference in the gaze-to-screen between the control layouts and between the experimental sessions (Figure 8). On overage, the participants looked at the visual feedback for 52.4% of the task time in both control layouts in the third session.

The outcome measures of completing the drinking task with the gamepad are included in Figure 7. The only tongue control condition that can be compared with the gamepad was the joystick-based layout and the with-screen condition because it contained a 2D joystick and the participants could see the buttons, similar to the gamepad. The paired *t*-test showed that the participant completed the drinking task using the gamepad with a significantly shorter task time [median 45.7 s vs. 70.2 s, *t*_(29)_ = −6.04, *p*< 0.000] and pause time [median 6.3 s vs. 26.2 s, *t*_(29)_ = −9.59, *p*< 0.000]. However, the tongue control required a lower number of commands [median 9 vs. 11, *t*_(29)_ = 2.34, *p*=0.026]. *T*-tests found no significant differences between the two control methods for moving time and trajectory length.

### 3.2. Button Task

**Figure 9:**
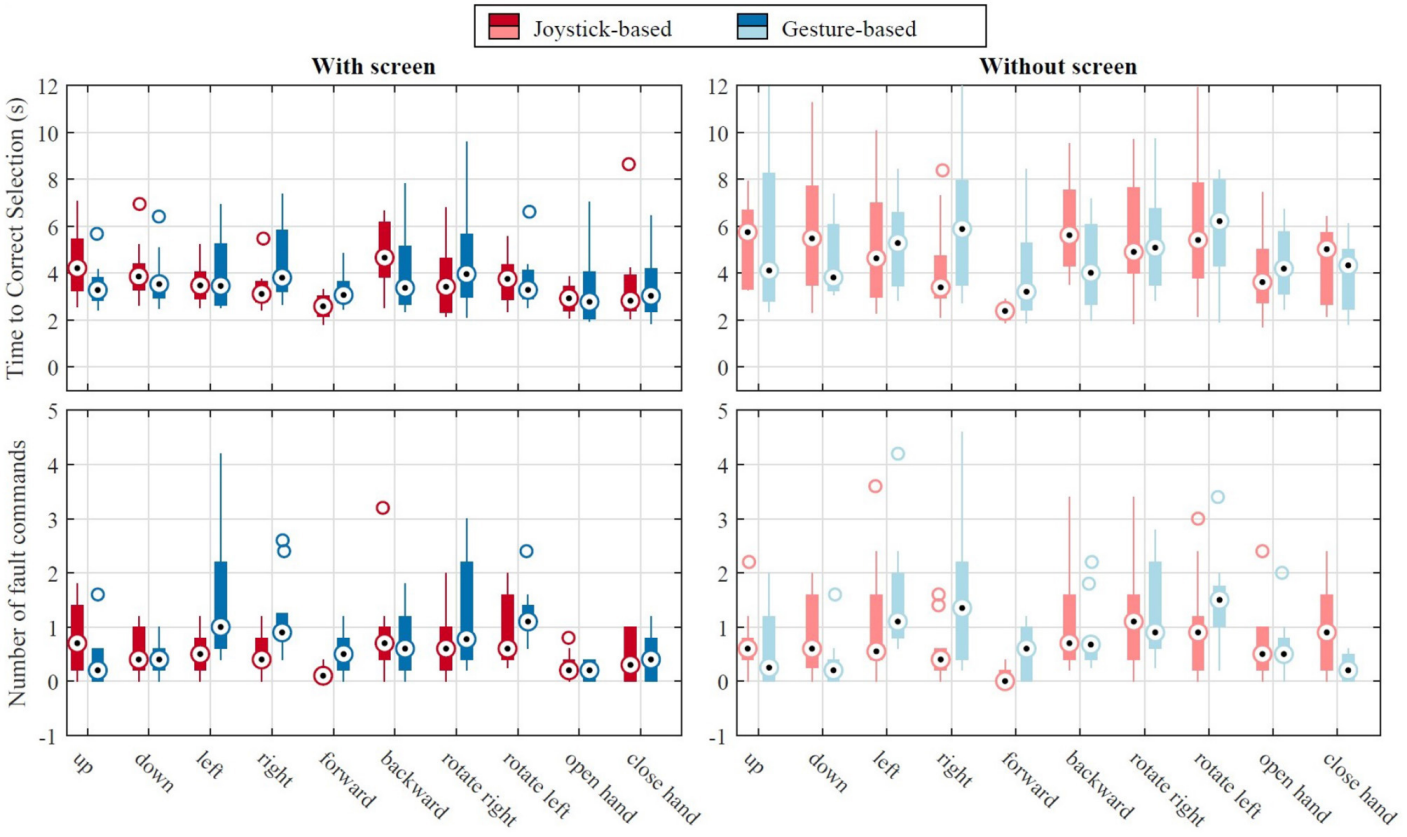
Results of the button task in the third session: time to correct selection of a command **(top)** and the number of fault commands **(bottom)** for the joystick-based and the gesture-based layouts. The “+” points are the outliers.

Both time-to-select and number of fault commands data were positively skewed and a log transfer did not result in normal distribution. Thus, we used the related-samples Friedman's analysis of variance by Ranks followed by pairwise Wilcoxon signed-rank test with a Bonferroni adjustment. The statistical tests did not show any significant difference between the time-to-select of the joystick-based and the gesture-based layouts, both in with-screen and without-screen conditions. Similar result was obtained for the number of fault commands. Using the joystick-based layout, it took 2.9 s (median) to select a target command with the screen and 3.1 s without it (including dwell time), and the difference was statistically significant (*Z* = − 3335.201, *p*< 0.000). Similarly, for the gesture-based layout, there was a significant difference (*Z* = −4.650, *p*< 0.000) between the time to select a target command with the screen (median 2.8 s) and without it (median 3.2 s). The number of fault commands increased by removing the screen only for the joystick-based layout (*Z* = −2.729, *p* = 0.006). Figure 9 shows the time-to-select and the number of fault command for each command. We can see a higher variability in the without screen data compared with screen.

### 3.3. Questionnaires

**Figure 10:**
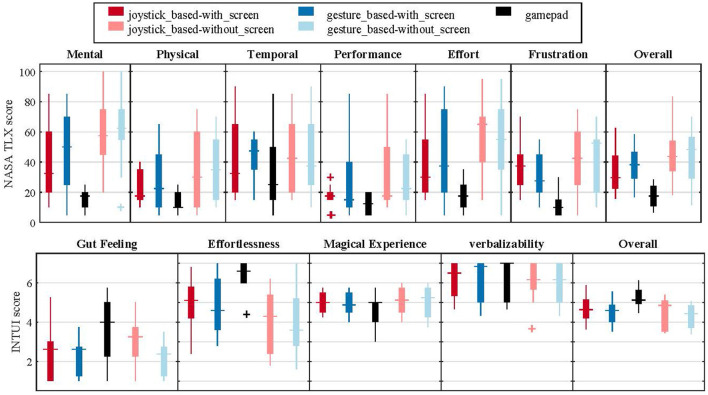
NASA TLX and INTUI scores. The “+” points are the outliers.

The participants rated the task load (NASA TLX) in different conditions similar to the pattern of the task time; i.e., the longer time to finish the task, the higher the task load (Figure 7 top left and Figure 10 top right). However, two-way ANOVA tests did not show any significant difference between the four control conditions with the ITCI system, neither for the NASA TLX nor the INTUI overall scores (Table 1). Controlling the EXOTIC Exo with the gamepad was rated significantly more intuitive (mean overall INTUI score 4.7 vs. 5.2) compared with the joystick-based layout with the screen [*t*_(9)_ = −5.81, *p*< 0.000]. The participants experienced lower task load (overall NASA TLX score) with the gamepad (mean 17.4) either than the ITCI (mean 32.7) with a significant difference [*t*_(9)_ = 4.14, *p* = 0.003].

Nine of the ten participants preferred to have the ITCI visual feedback while controlling the EXOTIC Exo. Seven participants preferred to use the gesture-based layout with the screen, while three chose the joystick-based. For the without-screen condition, six chose the gesture-based, and four chose the joystick-based layout.

### 3.4. Case Study With a User

**Figure 11:**
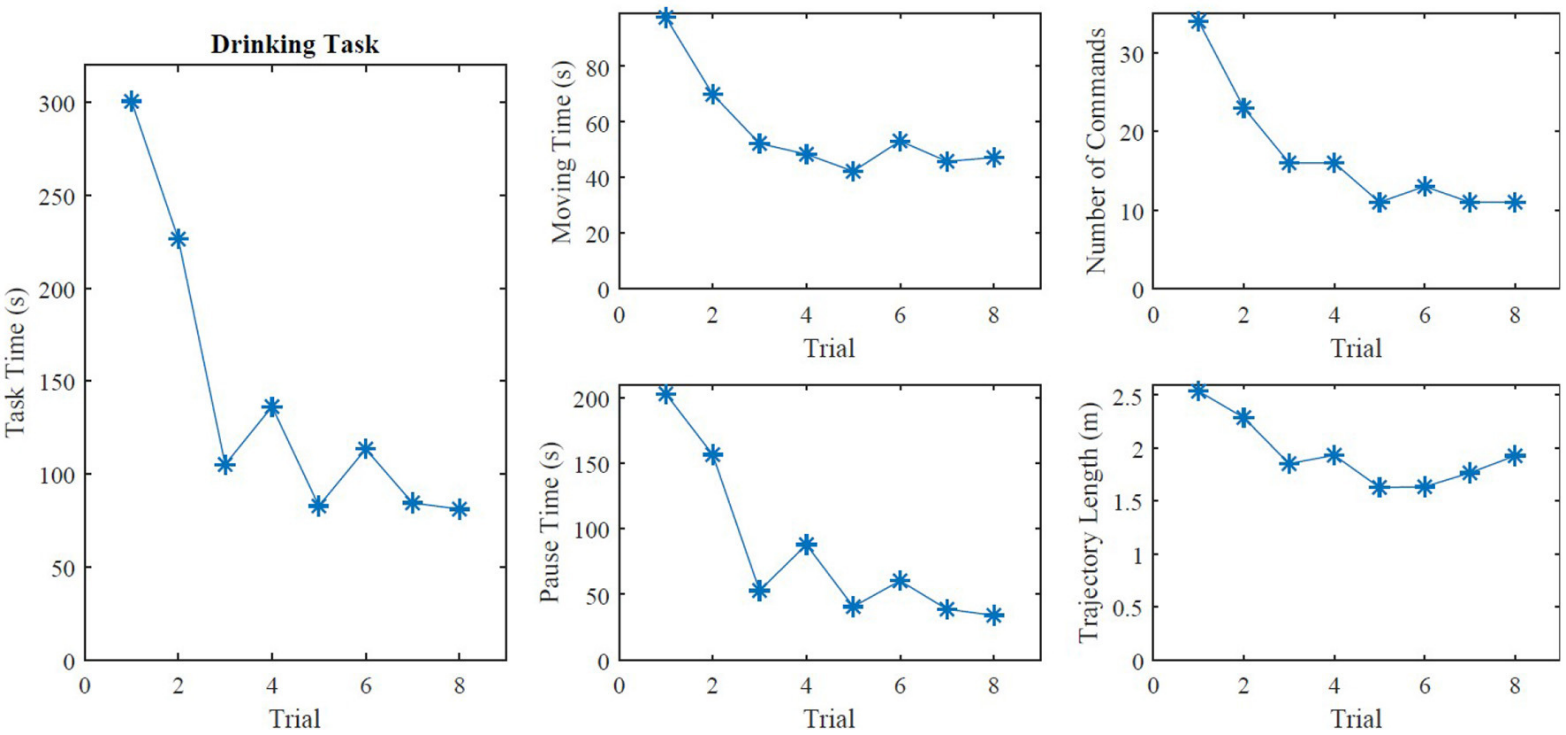
The outcome measures of the eight trials in the second session of the clinical case study.

The user controlled the EXOTIC Exo for the first time in the second session and we observed an improvement in all outcome measures over the trials (Figure 11). We compared the mean value of the last four trials of the user's second session with the median of the able-bodied group in the second session. The able-bodied participants performed four trials in the first session and four trials in the second session using the joystick-based control with the screen. The user completed the drinking task in 90.4 s while the able-bodied group finished it in 85.6 s; i.e., 5.6% slower than the able-bodied group. The moving time and pause time were 47.1 and 43.3 s for the user vs. 46.1 and 35.4 s for the able-bodied group respectively. On average, the user issued 11.5 commands to finish the drinking task, which was similar to the able-bodied group (11.4). The user's hand moved 1.74 m that is 18.0% less than the able-bodied group.

## 4. Discussion

This study demonstrated the feasibility of using a tongue interface (ITCI) to control a five-DOF arm exoskeleton and drink from a bottle (median task time 69.6 s for joystick-based control), which is a highly prioritized ADL by individuals with tetraplegia. The interface provided continuous and direct control of all five DOFs (ten commands) in one mode. Even if we tested the exoskeleton only for drinking, the interface affords full control of the exoskeleton for any arbitrary ADL as long as the exoskeleton workspace and physical constraint allow.

The results of using the gamepad for exoskeleton control provided a basis for between-study comparisons in the future. The gamepad control was 34.9% faster than the tongue control for drinking. This difference was due to the shorter pause time of the gamepad (6.3 vs. 26.2 s) as no significant difference was obtained between the moving times. Pause time represented the interval between two consecutive movements, consisting of a mental process for making a decision and then issuing a command. Considering the results of the button task including time-to-select (median 3.4 s) and the number of commands for the drinking task (median 9), time-to-select contributed the most to the difference between the gamepad and the tongue control. This was not surprising as the participants were more familiar with the gamepad, and further, the physical buttons afford a tactile feedback.

The study showed that it is possible to control the EXOTIC Exo to perform ADLs such as drinking with the ITCI without visual feedback on a screen. Removing the screen did not make a significant difference in task time with the gesture-based control. However, the joystick-based control was 31.2% faster with the screen. Many of the available robot interfaces for individuals with tetraplegia, such as the systems based on eye-tracking (Frisoli et al., 2012) or VEP (Sakurada et al., 2013), require concentration on a screen. However, removing the screen can improve the system's mobility and remove a redundant object from the user's surroundings. Further, avoiding the need of visual feedback allows to continuously monitor the hand and the object as in natural grasping, and it may provide a more efficient and safe control. We expect that with more training the performance of the tongue control without the screen will get closer to the with-screen condition, which should be tested in further studies.

In the case of controlling the EXOTIC Exo, which required ten commands, the participants performed similarly with the joystick-based and the gesture-based controls with no significant difference. In previous studies with the ITCI, the maximum number of buttons in the control layout was 18 equal to the number of inductive sensors (Andreasen Struijk et al., 2017a). On the contrary, the gesture-based control can accommodate up to 46 commands in a single mode (Mohammadi et al., 2020). Thus, gesture-based control can be more suitable for scenarios that require a higher number of commands. Furthermore, more participants preferred the gesture-based control scheme.

We recruited able-bodied participants since this study was the first for human users to test the EXOTIC Exo. This was a crucial step before individuals with tetraplegia could try the system. Able-bodied participants could immediately notify us if they felt any discomfort or pain and they could use the emergency stop button provided on the left wheelchair armrest (none of these cases happened). Since an SCI rarely affects the tongue functionality, we can expect similar outcomes from a study that includes participants with SCI. Different studies with the ITCI (Andreasen Struijk et al., 2017a) and another tongue interface (Kim et al., 2013) have shown a comparable performance between able-bodied participants and individuals with SCI. Furthermore, due to the gear mechanisms that drove the EXOTIC Exo joints, the participants could not move the exoskeleton through their arm muscles (the joints were not backdrivable). However, the soft glove allowed deliberate finger movement.

The clinical case study showed that an individual with tetraplegia could control the EXOTIC Exo for drinking and reached a similar performance to that of the able-bodied group. The drinking task completion time was only 5.6% longer for the user compared with the able-bodied participants. The interface provided full control for the user with no functionality in both arms on the contrary to interfaces that required some residual hand or finger volunteer movement (Tang et al., 2014; Kooren et al., 2016; Straathof et al., 2016; Hosseini et al., 2017). These interfaces can assist individuals who possess some residual arm movement capabilities in their arms. However, they are not applicable for individuals with complete tetraplegia. The proposed interface in this study provided continuous control contrary to the vocal control interface proposed by Gandolla et al. (2021) that only afforded discrete commands, which may compromise safety also due to unintended command from the environmental noise. In addition, the Itongue system has already been used by individuals with tetraplegia for computer and wheelchair control at home and ensures higher robustness and usability compared with interfaces based on surface EEG. Another essential feature of our tongue-ULE interface was accommodating all five DOF controls in one mode to avoid confusion due to mode switching (Herlant et al., 2016). The other tongue-ULE interface only controlled up to two DOFs (Ostadabbas et al., 2016; Zhang et al., 2021), which is insufficient for performing ADLs, except by incorporating automation or mode switching.

## 5. Conclusion

This study for the first time presented a tongue-based control of a five-DOF exoskeleton arm that provided continuous and direct control of all five DOFs in a single control mode and allowed the user to perform ADLs such as drinking without a screen for selecting commands. We compared a novel dynamic joystick-based control with a gesture-based control and did not find any significant differences between the task completion times in any of the conditions (with/without visual feedback). Furthermore, the study showed that removing the visual feedback from the gesture-based control scheme had no significant effect on the user's performance.

In addition, we demonstrated that an individual with no functional use of the arms due to severe tetraplegia could control the EXOTIC Exo and achieve a performance similar to that of the able-bodied participants. Future studies will include more individuals with tetraplegia and a longer intervention.

## Data Availability Statement

The raw data supporting the conclusions of this article will be made available by the authors, without undue reservation.

## Ethics Statement

The studies involving human participants were reviewed and approved by the North Denmark Region Committee on Health Research Ethics. The patients/participants provided their written informed consent to participate in this study. Written informed consent was obtained from the individual(s) for the publication of any potentially identifiable images or data included in this article.

## Author Contributions

MM, MT, SB, MGa, and LA developed the experiment setup and the software. KS, LA, and MT helped with developing the clinical study protocol and recruiting the user. MM, MT, and SB ran the experiment and collected the data, which were analyzed by MM, MT, HK, and LA. MM drafted and wrote the manuscript with input and supervision from HK and LA. All authors participated in the conception and design of the study and reviewed and approved the final manuscript.

## Funding

This study was a part of the interdisciplinary strategic project EXOTIC funded by Aalborg University, Denmark.

## Conflict of Interest

The authors declare that the research was conducted in the absence of any commercial or financial relationships that could be construed as a potential conflict of interest.

## Publisher's Note

All claims expressed in this article are solely those of the authors and do not necessarily represent those of their affiliated organizations, or those of the publisher, the editors and the reviewers. Any product that may be evaluated in this article, or claim that may be made by its manufacturer, is not guaranteed or endorsed by the publisher.

## Supplementary Material

Supplementary Video 1 A study participant controlling the upper-limb exoskeleton with the tongue interface without any visual feedback on the screen and completing the drinking task.
